# Emergency Department (ED) Walkouts in a Mental Health Crisis: West London NHS Trust Liaison Psychiatry Response to the London Metropolitan Police's Right Care Right Person Approach

**DOI:** 10.1192/bjo.2024.95

**Published:** 2024-08-01

**Authors:** Margherita Bielli, David Rosen, Sachin Patel, Yena Cho

**Affiliations:** 1 Imperial College Healthcare Trust, London, United Kingdom; 2 West London NHS Trust, London, United Kingdom

## Abstract

**Aims:**

Patients walking out of ED during mental health crises are commonly encountered in Liaison Psychiatry. Responsibility for high-risk or vulnerable walkouts had predominantly fallen on the police due to resource pressures in health and social care services. In 2023, London's Metropolitan Police announced a new partnership model, the “Right Care Right Person” (RCRP) approach. This supported the withdrawal of police involvement in mental health crises and allowed health and social care agencies who have the expertise and authority to act, to fulfil their role. This QI project aimed to understand the extent of police involvement in ED walkouts prior to the implementation of RCRP, introduce a new protocol for managing these situations, and evaluate its impact in terms of resource use and patient outcomes.

**Methods:**

The Trust's incident reporting system was used to identify mental health-related ED walkouts between May–August 2023, prior to the introduction of RCRP. Patients’ notes were reviewed to identify immediate actions taken following the walkout, including whether the police were involved, what action they took and patient outcomes. This was used to create a new Trust-wide ED walkout protocol, incorporating the Metropolitan Police's risk assessment tools. This was disseminated to frontline staff. A repeat analysis took place in November–December 2023, post-RCRP, to analyse how ED walkouts were being managed, and by which service. Furthermore, the analysis explored the nature of any patient harm which occurred following the incidents.

**Results:**

We found 29 walkouts from A&E between May–August 2023 (pre-RCRP), compared with 35 between November–December 2023 (post-RCRP). Police were called in 79% of cases pre-RCRP and 74% post-RCRP. Pre-RCRP police was not involved in 41% of cases, and in 81% of cases post-RCRP. Mental health services made first contact following walkout in 41% of cases pre-RCRP, and in 46% post-RCRP. LAS made contact in 29% of cases post-RCRP. Post-RCRP 26% of patients who walked out were admitted to a Mental Health Trust within 7 days. 20 patients had their treatment delayed, 5 suffered from neglect. 3 patient walkouts resulted in harm to others, and 2 resulted in self-harm.

**Conclusion:**

As expected, police responded to fewer walkout reports, and our data shows this gap has been filled by other services. The Trust's risk assessment-based approach to managing walkouts has shown promising results. The next stage of the project will focus on developing local protocols for the identification and management of patients at high risk of walkout.

